# Cognitive decline in U.S. military veterans: risk factors and clinical implications

**DOI:** 10.3389/frdem.2026.1704367

**Published:** 2026-02-03

**Authors:** Rob J. MacLennan, Benjamin A. Chapin, Laurence M. Solberg, David J. Clark

**Affiliations:** 1 Geriatric Research, Education, and Clinical Center, North Florida/South Georgia Veterans Health System, Gainesville, FL, United States; 2 Department of Neurology, University of Florida College of Medicine, Gainesville, FL, United States; 3 Perioperative Cognitive Anesthesia Network, Department of Anesthesiology, University of Florida College of Medicine, Gainesville, FL, United States; 4 Division of Behavioral Neurology and Neuropsychiatry, Department of Neurology, University of Florida College of Medicine, Gainesville, FL, United States; 5 University of Central Florida College of Medicine, Orlando, FL, United States; 6 University of Florida College of Nursing, Gainesville, FL, United States; 7 Brain Rehabilitation Research Center, North Florida/South Georgia Veterans Health System, Gainesville, FL, United States

**Keywords:** diabetes, hearing impairment, chronic pain, smoking, depression, social isolation, hypercholesterolemia, sleep disturbance

## Abstract

Military veterans have higher aggregate prevalence of risk factors for cognitive decline than non-veterans. This includes risk factors like diabetes, chronic pain, smoking, depression, and more. The disparity in prevalences is due in part to the unique experiences and exposures of their military service. Alzheimer's disease and other dementias are debilitating diseases with large financial and logistical burdens. These burdens are held by the patient, their family, friends, and caregivers, as well as healthcare professionals, and healthcare systems. Standardized screening for these risk factors may be helpful for understanding risk profiles that lead to cognitive decline. Additionally, screening must occur early to encourage early intervention and behavioral modifications and to reduce these burdens. This perspective presents the prevalence of risk factors for cognitive decline in the Veteran and non-veteran populations and proposes an approach to managing risk factors in Veterans.

## Introduction

US military service members have experiences and exposures that are different from those of the general population. Many perform hazardous and stressful work, work in harsh environments, and live in geographically isolated locations. As a result, US military service veterans (Veterans) have greater prevalence of many health risk factors than non-veterans. This may put Veterans at greater risk of developing diseases than non-veterans, including Alzheimer's disease and related dementias (ADRD; Powell et al., 2023). In the United States, ADRD is one of the costliest chronic disease categories (2024 Alzheimer's disease facts and figures, 2024). From disease onset to death, the estimated total cost per individual is $400 thousand, 70% of which is borne by caregivers for unpaid caregiving, medications, and home health support with the remaining being paid by Medicare and Medicaid (2024 Alzheimer's disease facts and figures, 2024; Skaria, 2022). Between the year 2018 and 2050, nationwide costs are projected to add up to $47 trillion (Nandi et al., 2022). These costs impact the individual and their family, as well as institutions that provide care such as the US Veterans Health Administration (VHA).

The search for articles was initiated by reviewing the current, comprehensive articles by The Lancet and the Alzheimer's Association (2024 Alzheimer's disease facts and figures, 2024; Livingston et al., 2024). Next, the search was expanded by database searches through Google Scholar and PubMed with the terms (veteran OR military OR “general population”) AND (Alzheimer's OR dementia OR ADRD or “cognitive decline”) AND (“risk factors”). The search was then narrowed by using terms for specific risk factors which included diabetes, hearing impairment, chronic pain, smoking, depression, social isolation, hypercholesterolemia, sleep disturbance, alcohol consumption, post-traumatic stress disorder, hypertension, obesity, physical inactivity, education level, traumatic brain injury, and air pollution. Risk factors were included based on the strength of evidence, and their interest to the military and Veteran populations (Goldstein et al., 2025; Livingston et al., 2024; Mannes et al., 2022; Veitch et al., 2013). The search was further narrowed by variations of terms for risk factors (e.g., hypertension or high blood pressure, sleep disturbance or sleep apnea or OSA). Date ranges included 2020-present, 2015-present, 2010-present, etc. with preference for more recent articles.

Evidence of elevated risk factors in Veterans and the effect on the likelihood of developing ADRD is widely dispersed in the literature. Thus, consolidating this evidence is foundational to improving the care and outcomes in Veterans.

## Prevalence of risk factors in veterans and non-veterans

There are many known risk factors for the development of ADRD such as smoking, excessive alcohol consumption, hypertension, and sleep disturbance (Livingston et al., 2024). For some risk factors, there is a notable heightened prevalence in Veterans, while for other risk factors there is little or no known difference (Table 1; Littman et al., 2013; Ogden et al., 2020; Washington, 2022). Even in cases where there is little difference, the relative cost to the VHA may be higher. This is because the prevalence of some risk factors is higher in Veterans who receive care through VHA, as opposed to other sources such as private insurance (Wisco et al., 2022). The following subsections include relevant risk factors, roughly ordered by disparity of prevalence among Veterans and non-veterans. Where scientific literature is inconclusive, we have done our best to objectively present the strength of the evidence.

**Table 1:** Summary of prevalence for cognitive decline risk factors in veteran and non-veteran populations.

**Risk factor**	**Age range**	**VHA vs non-VHA**	**Prevalence, unadjusted % (group)**	**Prevalence, adjuste**	**Cognitive decline effect estimate**	**Study design**	**Population**
**Diabetes** **Effect estimate: HR 1.90 (**<**50 years; Qi et al., 2024); RR 1.43 (**Xue et al., 2019**)** **Veterans**							
Liu et al. (2017)	22–65+	non-VHA	24	Not available	Not available	Pooled, serial, cross-sectional survey	US Veterans
Jackson et al. (2015)	<50–70 (μ = 53.6)	VHA	20	Not available	Not available	Retrospective observational analysis	US Veterans, BMI 30+ OR BMI 25+ with a weight-related disorder
**Non-veterans**							
Wang et al. (2021)	18–65+ (μ = 48.2)	non-VHA	14.6	14.3 (age)	Not available	Pooled, serial, cross-sectional survey	US general population
Benoit et al. (2019)	18–79	non-VHA	Not available	10.5 (age)	Not available	Pooled, serial, cross-sectional survey	US general population
**Hearing impairment** **Effect estimate: HR 1.37 (Livingston et al., 2024); RR 1.90 (Livingston et al., 2020)** **Veterans**							
Lucas and Zelaya (2020)	18+ 18–44 45–64 65–74	non-VHA	19.8 (18–44) 27.7 (45–64) 42.8 (65–74)	27.1 (age, 18+)	Not available	Cross-sectional survey	US Veterans
**Non-veterans**							
National Institute on Deafness and Other Communication Disorders (2024)	45–54 55–64 65–74	non-VHA	5 (45–54) 10 (55–64) 22 (65–74)	Not available	Not available	Report	US general population
Madans et al. (2021)	18+	non-VHA	14.6	Not available	Not available	Cross-sectional survey	US general population
Lucas and Zelaya (2020)	18+ 18–44 45–64 65–74	non-VHA	5.7 (18–44) 18.6 (45–64) 37.2 (65–74)	16 (age, 18+)	Not available	Cross-sectional survey	US general population
**Chronic pain** **Effect estimate: HR 1.08–1.50 (Tian et al., 2023)** **Veterans**							
Taylor et al. (2024)	Not available (18+)	non-VHA	Any: 49.2–64.8 Multiple: 20.8–35.5	Not available	Not available	Pooled, serial, cross-sectional survey	US Veterans
Mannes et al. (2022)	18–65+	VHA & non-VHA	32.93 45.26 (VHA) 26.88 (non-VHA)	Not available	Not available	Cross-sectional survey	US Veterans
Zelaya et al. (2020)	20–65+	non-VHA	31.5	Not available	Not available	Cross-sectional survey	US Veterans
**Non-veterans**							
Taylor et al. (2024)	Not available (18+)	non-VHA	Any: 45.9–54.8 Multiple: 21.6–28.9	Not available	Not available	Pooled, serial, cross-sectional survey	US general population
Nahin et al. (2023)	18–50+ (μ = 49)	non-VHA	20.8	Not available	Not available	Cohort, longitudinal	US general population
Rikard et al. (2023)	18–85+	non-VHA	20.9	19.7 (age)	Not available	Serial, cross-sectional survey	US general population
Mannes et al. (2022)	18–65+	VHA & non-VHA	20	Not available	Not available	Cross-sectional survey	US general population
Zelaya et al. (2020)	20–65+	non-VHA	20.1	Not available	Not available	Cross-sectional survey	US general population
**Cigarette smoking and other tobacco use** **Effect estimate: HR 1.88 (Tsao et al., 2023); RR 1.30 (Zhong et al., 2015)** **Veterans**							
Nieh et al. (2021)	17–45+	non-VHA	20.1	Not available	Not available	Prospective cohort	US Veterans
Odani et al. (2018)	18–50+	non-VHA	29.2	Not available	Not available	Pooled, serial, cross-sectional survey	US Veterans
Brown (2010)	18+	non-VHA	Not available	27 (age, men) 23 (age, women)	Not available	Pooled, serial, cross-sectional survey	US Veterans
**Non-veterans**							
Phillips et al. (2017)	18–65+	non-VHA	Not available	25.2 (males) 15.4 (females)	Not available	Cross-sectional survey	US general population
Brown (2010)	18+	non-VHA	Not available	21 (age) 21 (age, men) 18 (age, women)	Not available	Pooled, serial, cross-sectional survey	US general population
**Depression** **Effect estimate: HR 2.41 (Elser et al., 2023); RR 2.25 (Livingston et al., 2024)** **Veterans**							
Finnegan and Randles (2023)	16–99 (μ = 62)	UK NHS	17.8	22.6 (ages 28–37) 28.6 (ages 38–47)	Not available	Retrospective, cohort, medical records	UK Veterans
Moradi et al. (2021)	(μ = 13–70)	VHA & non-VHA	20	Not available	Not available	Meta-analysis, cross-sectional studies	Multi-national Veterans
Gould et al. (2015)	50+	non-VHA	11	Not available	Not available	Cross-sectional survey	US Veterans, men
Hankin et al. (1999)	(μ = 62)	VHA	31	29	Not available	Prospective, longitudinal cohort	US Veterans, male
**Non-veterans**							
Substance Abuse Mental Health Services Administration (2022)	18+	non-VHA	8.3 (past year)	Not available	Not available	Cross-sectional survey	US general population
Daly et al. (2021)	18–65+ (μ = 48)	non-VHA	8.9 (pre-pandemic) 14.2 (April 2020)	8.7 (pre-pandemic) 14.4 (April 2020; age, sex, race/ethnicity, education, income)	Not available	Longitudinal, cross-sectional survey	US general population
Gould et al. (2015)	50+	non-VHA	12.8	Not available	Not available	Cross-sectional survey	US general population, men
**Social isolation** **Effect estimate: HR 1.38–2.34 (Saczynski et al., 2006); RR 1.41–1.57 (Kuiper et al., 2015)** **Veterans**							
Vespa (2023)	18+, 65+	non-VHA	34.4 (18+) 42.8 (65+)	Not available	Not available	Cross-sectional, descriptive analysis	US Veterans
**Non-veterans**							
Vespa (2023)	18+, 65+	non-VHA	26.5 (18+) 45.6 (65+)	Not available	Not available	Cross-sectional, descriptive analysis	US general population
**Hypercholesterolemia** **Effect estimate: HR 1.33 (Livingston et al., 2024); ES 2.01 (Wee et al., 2023)** **Veterans**							
Nguyen et al. (2023)	18+ (μ = 62)	VHA	37.8	Not available	Not available	Retrospective cohort, observational	US Veterans
Washington (2022)	18–85+	VHA	47.3	Not available	Not available	Observational, cross-sectional analysis	US Veterans
**Non-veterans**							
Tsao et al. (2023)	20+	non-VHA	Not available	34.7	Not available	Annual systematic review	US general population
**Sleep disturbance** **Effect estimate: HR 1.51 (Wong and Lovier, 2023); RR 1.20–1.50 (Shi et al., 2018)** **Veterans**							
Goldstein et al. (2025)	18–75+	non-VHA	21 (sleep apnea, all) 23 (sleep apnea, men)	Not available	Not available	Cross-sectional survey	US Veterans
Faestel et al. (2013)	21–75+	non-VHA	22.4	22.4 (age, sex, race/ethnicity)	Not available	Cross-sectional survey	US Veterans
**Non-veterans**							
Goldstein et al. (2025)	18–75+	non-VHA	9 (sleep apnea, all) 13 (sleep apnea, men)	Not available	Not available	Cross-sectional survey	US general population
Faestel et al. (2013)	21–75+	non-VHA	27.9	20.1 (age, sex, race/ethnicity)	Not available	Cross-sectional survey	US general population
**Alcohol consumption (heavy)** **Effect estimate: HR 1.08 (Jeon et al., 2023); RR 1.18 (Livingston et al., 2020)** **Veterans**							
Teeters et al. (2017)	Not specified	VHA & non-VHA	7.5 (heavy)	Not available	Not available	Review	US Veterans
Wagner et al. (2007)	17–75+	non-VHA	7.5	Not available	Not available	Pooled analysis, repeated, cross-sectional survey	US Veterans
**Non-veterans**							
Teeters et al. (2017)	Not specified	VHA & non-VHA	Not available	6.5 (comparable non-veterans)	Not available	Review	US general population
Wagner et al. (2007)	17–75+	non-VHA	Not available	6.5 (comparable non-veterans)	Not available	Pooled analysis, repeated, cross-sectional survey	US general population
**Post-traumatic stress disorder** **Effect estimate: HR 1.61–2.11 (Günak et al., 2020); OR 2.20 (Qureshi et al., 2010)** **Veterans**							
Eibner et al. (2016)	Not available	VHA & non-VHA	3.3	Not available	Not available	Cohort-based, modeling	US Veterans
Schnurr et al. (2009)	18–65+	VHA & non-VHA	23 (VHA) 7 (non-VHA)	Not available	Not available	Review	US Veterans
**Non-veterans**							
Schnurr et al. (2009)	18–65+	VHA & non-VHA	6	Not available	Not available	Review	US general population
**Hypertension** **Effect estimate: HR 1.49–2.00 (****Livingston et al., 2020****; Tsao et al.**, 2023**); RR 1.61 (Norton et al., 2014)** **Veterans**							
Yamada et al. (2023)	(μ = 66)	VHA	71 (140/90) 81 (130/90) 87 (130/80)	Not available	Not available	Retrospective cohort	US Veterans
Washington (2022)	Not available	VHA	48.2	Not available	Not available	Comparative analysis, longitudinal, cross-sectional data	US Veterans
Boersma et al. (2021)	25+ 25–64 65+	non-VHA	Not available	32.2 (25–64, male, Veteran) 25.6 (25–64, female, Veteran) 64.9 (65+, male, Veteran) 69.8 (65+, female, Veteran)	Not available	Pooled report, cross-sectional survey	US Veterans
**Non-veterans**							
Centers for Disease Control and Prevention (2023)	18+	non-VHA	48.1	Not available	Not available	Survey, guideline application	US general population
Washington (2022)	Not available	non-VHA	45	Not available	Not available	Comparative analysis, longitudinal, cross-sectional data	US general population
Boersma et al. (2021)	25+ 25–64 65+	non-VHA	Not available	26.6 (25–64, male, non-veteran) 22.4 (25–64, female, non-veteran) 62.4 (65+, male, non-veteran) 61.9 (65+, female, non-veteran)	Not available	Pooled report, cross-sectional survey	US general population
**Obesity** **Effect estimate: HR 1.34 (Ma et al., 2020); RR 1.60 (Barnes and Yaffe, 2011)** **Veterans**							
Breland et al. (2017)	Not available	non-VHA	41	Not available	Not available	Descriptive analysis, cross-sectional	US Veterans
**Non-veterans**							
Hales et al. (2020)	20+	non-VHA	42.5 43.0 (men) 42.1 (women)	42.4 (age)	Not available	Pooled analysis, serial, cross-sectional survey	US general population
Ogden et al. (2020)	20+ 20–74	non-VHA	42.5	42.4 (20+) 42.8 (20–74)	Not available	Stratified, multistage probability, cross-sectional survey	US general population
Breland et al. (2017)	Not available	non-VHA	38	Not available	Not available	Descriptive analysis, cross-sectional	US general population
Flegal et al. (2016)	20+ (μ ≈ 47)	non-VHA	37.9 35.2 (men) 40.5 (women)	37.7 (age)	Not available	Pooled analysis, serial, cross-sectional survey	US general population
**Physical inactivity** **Effect estimate: HR 1.40 (Kivimäki et al., 2019); RR 1.30–1.82 (Norton et al., 2014**; Yan et al.**, 2020)** **Veterans**							
Littman et al. (2013)	21–85+	non-VHA	Not available	49 30.8 (inactive) 18.2 (insufficiently active) (age, demographics)	Not available	Stratified, multistage probability, cross-sectional survey	US Veterans, male
Hoerster et al. (2012)	18–65+	non-VHA	24.6	Not available	Not available	Population-based, cross-sectional survey	US Veterans, male
**Non-veterans**							
Littman et al. (2013)	21–85+	non-VHA	Not available	56.1 32.5 (inactive) 23.6 (insufficiently active) (age, demographics)	Not available	Stratified, multistage probability, cross-sectional survey	US general population, male
Hoerster et al. (2012)	18–65+	non-VHA	21.5	Not available	Not available	Population-based, cross-sectional survey	US general population, male
**Education level** **Effect estimate: HR 1.76 (Sabia et al., 2018); RR 1.59 (Barnes and Yaffe, 2011)** **Veterans**							
Chakrabarti et al. (2023)	25–69	non-VHA	Not available	27 (Bachelor's or higher)	Not available	Comparative analysis, survey	US Veterans, male
Rolen (2017)	18+	non-VHA	30.5 (Bachelor's or higher)	Not available	Not available	Descriptive analysis, survey	US Veterans
**Non-veterans**							
Chakrabarti et al. (2023)	25–69	non-VHA	Not available	34 (Bachelor's or higher)	Not available	Comparative analysis, survey	US general population, male
Rolen (2017)	Not available	non-VHA	31.1 (Bachelor's or higher)	Not available	Not available	Descriptive analysis, survey	US general population
**Traumatic brain injury** **Effect estimate: HR 2.36–3.77 ((Barnes et al., 2018)); RR 1.66 (Gardner et al., 2023)** **Veterans**							
Kornblith et al. (2020)	65+ (μ = 75.2)	non-VHA	7.76	Not available	Not available	Longitudinal cohort, survey	US Veterans
Gardner et al. (2023)	(μ = 49–81)	VHA & non-VHA	35 36 (males) 3.5 (females)	Not available	RR = 2.13 (113%), 2.13 (113%, males), 2.13 (113%, females)	Systematic review, meta-analysis	US Veterans
Kornblith et al. (2020)	51+	non-VHA	Not available	36	Not available	Nationally representative survey	US Veterans, male
**Non-veterans**							
Kornblith et al. (2020)	65+ (μ = 75.2)	non-VHA	8.97	Not available	Not available	Longitudinal cohort, survey	US general population
Gardner et al. (2023)	(μ = 49–81)	VHA & non-VHA	31 43 (males) 22 (females)	Not available	RR = 1.52 (52%), 2.07 (107%, males), 1.43 (43%, females)	Systematic review, meta-analysis	US general population
Kornblith et al. (2020)	51+	non-VHA	Not available	45	Not available	Nationally representative survey	US general population, male
**Air pollution and toxic exposures** **Effect estimate: HR 1.45 (burn pits, Wilker et al., 2023), 1.68 (Agent Orange, Martinez et al., 2021)** **Veterans**							
Chari et al. (2024)	Not available	VHA & non-VHA	80 (Burn pits; OEF, OIF, & OND)	Not available	Not available	Policy analysis and synthesis	US Veterans
Martinez et al. (2021)	≈ 45–85 (μ = 62)	VHA	12.2 (Agent Orange; Vietnam War)	Not available	HR = 1.68 (68%)	Longitudinal cohort	US Veterans

### Diabetes

Type 2 diabetes is characterized by hyperglycemia due to insulin deficiency (Smushkin and Vella, 2010). Estimates in the non-veteran population find that 8%−14% have diabetes (Benoit et al., 2019; Wang et al., 2021). This contrasts with Veterans, of whom 20%−24% are affected (Jackson et al., 2015; Liu et al., 2017), suggesting Veterans are approximately twice as likely to have diabetes as non-veterans. This prevalence of diabetes is particularly concerning, as diabetes increases the risk of ADRD by up to 90% (Qi et al., 2024; Xue et al., 2019).

### Hearing impairment

Hearing impairment can be diagnosed by a pure tone average of 40 decibels or more at frequencies of 500, 1000, 2000, and 4000 Hz (Taljaard et al., 2016). In ages 45–64, prevalence of hearing loss in non-veterans is estimated to be between 5 and 17% (Lucas and Zelaya, 2020; Madans et al., 2021; National Institute on Deafness and Other Communication Disorders, 2024). In Veterans between the ages of 45–64 however, hearing loss is estimated to affect 24% of the population (Lucas and Zelaya, 2020). In those 65–74 years old, the disparity remains with 22%−37.2% of non-veterans, and 48.2% of Veterans experiencing hearing loss (Lucas and Zelaya, 2020; Madans et al., 2021; National Institute on Deafness and Other Communication Disorders, 2024). Hearing loss increases the likelihood of developing dementia by approximately 37% (Livingston et al., 2024).

### Chronic pain

Chronic pain is defined as pain that persists or recurs for more than 3 months (Treede et al., 2019). It is estimated that 20%−55% of non-veterans have experienced chronic pain compared to 32%−65% of Veterans (Nahin et al., 2023; Rikard et al., 2023; Taylor et al., 2024; Zelaya et al., 2020). Further, 22%−29% of non-veterans, and 21%−36% of Veterans have had multiple sites with chronic pain (Taylor et al., 2024). Within the Veteran population, 45% of Veterans who receive healthcare from the VHA had chronic pain, while only 27% of Veterans who receive their healthcare elsewhere had chronic pain (Mannes et al., 2022). This is an important consideration as the number of chronic pain sites has been found to increase risk of dementia in the range of 8%−40% (Tian et al., 2023).

### Cigarette smoking and other tobacco use

A “smoker” is defined as one who currently smokes and has smoked at least 100 cigarettes in their lifetime (Agaku et al., 2014). Among non-veterans, 15%−21% are smokers (Brown, 2010; Phillips et al., 2017), while 22%−30% of Veterans are smokers (Nieh et al., 2021; Odani et al., 2018), with one study reporting up to 50% of Veterans are smokers (Odani et al., 2018). Among Veterans aged 18–25, 57% engage in some form of tobacco use (Odani et al., 2018). Additionally, 57% of non-veterans never smoked while 33% of Veterans never smoked (Nieh et al., 2021). The greater prevalence of smoking and tobacco use among Veterans is concerning because this risk factor increases the risk of developing ADRD by 30%−90% (Tsao et al., 2023; Zhong et al., 2015).

### Depression

Depression is generally considered to be present when depressive symptoms last 2 weeks or more with significant social degradation (Rondón Bernard, 2018). Approaches for determining the prevalence of depression are not uniform, which results in a range of estimates. In non-veterans, the reported prevalence of depression ranges from 9 to 19% (Daly et al., 2021; Substance Abuse Mental Health Services Administration, 2022) and in Veterans it ranges from 11 to 31% (Finnegan and Randles, 2023; Gould et al., 2015; Hankin et al., 1999; Moradi et al., 2021). For major depressive disorder, the prevalence reported in the literature is 8% for non-veterans and 13% for Veterans (Gould et al., 2015; Substance Abuse Mental Health Services Administration, 2022). Those with a history of depression have a 240% increase in risk for developing ADRD, making it a highly consequential risk factor (Elser et al., 2023; Livingston et al., 2024).

### Social isolation

Social isolation is when a person objectively lacks or has limited social contact with others (Donovan and Blazer, 2020). Among all ages, the US Census Bureau reports that 27% of non-veterans and 34% of Veterans experience social isolation (Vespa, 2023). Social isolation increases with age, but the gap closes in ages 65+ with 46% of non-veterans and 43% of Veterans being isolated (Kannan and Veazie, 2023; Vespa, 2023). Social isolation is associated with a 30%−65% increase in the risk for dementia (Kuiper et al., 2015; Penninkilampi et al., 2018; Saczynski et al., 2006).

### Hypercholesterolemia

Hypercholesterolemia is defined as having total cholesterol of greater than 200 mg/dL (Civeira et al., 2022). Among all ages, 35% of non-veterans and 38% of Veterans have high cholesterol (Nguyen et al., 2023; Tsao et al., 2023). However, within the VHA, 47% of Veterans have high cholesterol (Washington, 2022). High cholesterol is associated with an estimated 30% increase in the risk of dementia (Livingston et al., 2024) and a 100% increase in the risk of mild cognitive impairment (MCI; Wee et al., 2023).

### Sleep disturbance

Sleep disturbance includes insomnia, obstructive sleep apnea, sleep-related movement disorder, and more (Livingston et al., 2020; Shi et al., 2018). Veterans experience sleep disturbances more than non-veterans (Alexander et al., 2016; Fung et al., 2013; Mustafa et al., 2005). One study estimates the prevalence in non-veterans and Veterans is 20 and 23% respectively (Faestel et al., 2013). It has been reported that twice as many Veterans (21%) experience obstructive sleep apnea compared to non-veterans (9%; Goldstein et al., 2025). Sleep disturbance is associated with an increased risk of ADRD by 20%−50% (Shi et al., 2018; Wong and Lovier, 2023).

### Alcohol consumption

The US National Institute on Drug Abuse reports the prevalence of alcohol use among non-veterans is 51%, while in military Veterans it is 57% (Teeters et al., 2017). Further, heavy alcohol use, defined as consuming 5 or more drinks on 5 or more days in the past month, was 6.5% among non-veterans and 7.5% among Veterans (Teeters et al., 2017; Wagner et al., 2007). The relationship between alcohol consumption and cognitive decline is complex, as mild, and moderate alcohol consumption are found to reduce the risk of dementia by 21 and 7% respectively (Jeon et al., 2023). However, heavy alcohol consumption increases risk by 8%−18% (Jeon et al., 2023; Livingston et al., 2020; Sabia et al., 2018).

### Post-traumatic stress disorder

Post-traumatic stress disorder (PTSD) results from experiencing or witnessing a traumatic event, with symptoms that interfere with one's daily life. Six percent of non-veterans and 7% of Veterans have experienced PTSD (Schnurr et al., 2009). However, PTSD in Veterans likely results in relatively greater costs to VHA because even though only 7% of Veterans experience PTSD, it is estimated that up to 23% of Veterans who receive healthcare through VHA have had PTSD (Schnurr et al., 2009). In fact, Veterans with PTSD are 13.5 times more likely to be diagnosed than non-veterans who have PTSD (Eibner et al., 2016). PTSD is associated with 50%−100% increased risk of ADRD (Bhattarai et al., 2019; Günak et al., 2020; Qureshi et al., 2010; Yaffe et al., 2010).

### Hypertension

The criteria for hypertension varies depending on source, with lenient criteria being systolic blood pressure (SBP) ≥140 mmHg or diastolic blood pressure (DPB) ≥90 mmHg (Carretero and Oparil, 2000), and more strict criteria including SBP ≥130 mmHg, DPB ≥80 mmHg, or hypertensive drug prescription (Yamada et al., 2023). Some studies report that approximately 48% of non-veterans and Veterans have hypertension (Boersma et al., 2021; Centers for Disease Control and Prevention, 2023; Washington, 2022). One study that utilized the most strict criteria found that 87% of Veterans have hypertension (Yamada et al., 2023). Uncontrolled hypertension is common despite efforts to reduce it, and has been found to increase the risk of dementia by 50%−100% (Livingston et al., 2020; Norton et al., 2014; Tsao et al., 2023).

### Obesity

Obesity is defined as having a body mass index of 30 kg/m^2^ or more (Chooi et al., 2019). In ages 20–39, 32% of non-veterans are obese and among all ages 38%−43% are obese (Breland et al., 2017; Flegal et al., 2016; Hales et al., 2020; Ogden et al., 2020). Studies of the Veteran population report that 46% of those ages 18–44 are obese and overall 41% are obese (Breland et al., 2017). Obesity increases the risk of dementia by 34% (Ma et al., 2020).

### Physical inactivity

CDC physical activity guidelines recommend 150 min of moderate intensity, or 75 min of vigorous activity per week (Littman et al., 2013). Failing to meet these guidelines is considered physical inactivity (Thivel et al., 2018). Lack of physical activity is common, with 22%−33% of non-veterans and 25%−31% of Veterans reporting being (Hoerster et al., 2012; Littman et al., 2013). Further, 24% of non-veterans and 18% of Veterans reported being active for more than 10 min per day, but less than CDC guidelines (Littman et al., 2013). The relationship between physical inactivity and cognition can vary depending on how they're quantified, but trials have reported improvements in cognition following exercise interventions (Carta et al., 2021; Sáez De Asteasu et al., 2017). Studies report that physical inactivity increases the risk of dementia by 30%−82% (Kivimäki et al., 2019; Norton et al., 2014; Yan et al., 2020).

### Education level

Both the non-veteran and Veteran populations have high rates of high school graduation (Department of Defense, 2023; Heckman and LaFontaine, 2010; National Center for Education Statistics, 2024), and this acts as a ceiling effect for educational attainment as a risk factor (Sharp and Gatz, 2011). Further, evidence suggests comparable attainment of undergraduate degrees with 9% of non-veterans and 13% of Veterans having earned an associate's degree (Rolen, 2017), and 34% of non-veterans and 27% of Veterans earning a Bachelor's degree or higher (Chakrabarti et al., 2023). The effect of low educational attainment on the development of ADRD varies depending on the criteria, with studies reporting very different odds depending on variables such as years of education, demographics, student-to-teacher ratio, and more (Barnes and Yaffe, 2011; Clouston et al., 2020; Crimmins et al., 2018; Sabia et al., 2018; Soh et al., 2023).

### Traumatic brain injury

Traumatic brain injury (TBI) can be categorized by a range of severities from mild to severe (Livingston et al., 2020). The prevalence of TBI is complicated due to underreporting of mild TBI and variable diagnosis (Feigin et al., 2013). Studies report that TBI prevalence in non-veterans is 31% while in Veterans it is 35% (Gardner et al., 2023). These figures include males and females together. Separated by sex, 43% of male and 22% of female non-veterans, and 36% of male and 3.5% of female Veterans have TBI (Gardner et al., 2023). The difference between sexes in the Veteran population is possibly influenced by females being ineligible for combat roles and some combat training until 2015. Thus, prevalence in male non-veterans (45%) and Veterans (36%; Gardner et al., 2023; Kornblith et al., 2020) may be the most representative figures. Evidence suggests that mild TBI may increase risk of dementia by as much as 100% (Barnes et al., 2018; Walker et al., 2022). Moderate and severe TBI have been found to increase risk of dementia by 100%−300% (Plassman et al., 2000; Walker et al., 2022).

### Air pollution and toxic exposures

Military activity can result in exposure to exhaust, pesticides, and heavy metals (DeBeer et al., 2017; Skalny et al., 2021) to a greater degree than what non-veterans are exposed to (Hoisington et al. 2024). Two of the most conspicuous exposures are Agent Orange and burn pits. Agent Orange is an herbicide used by the U.S. during the Vietnam War and was not used in the US (Martinez et al., 2021). An estimated 12% of Vietnam Veterans were exposed to Agent Orange (Martinez et al., 2021). Burn pits were used during Operations Enduring Freedom, Iraqi Freedom, and New Dawn to dispose of trash and human waste, often lit using jet or diesel fuel (Trembley et al., 2024). Eighty percent of Veterans deployed in these operations report being exposed to burn pits (Chari et al., 2024). Those exposed to Agent Orange and burn pits are at greater risk of cognitive decline. Veterans exposed to Agent Orange are 68% more likely to be diagnosed with dementia (Martinez et al., 2021). The risk associated with burn pit exposure varies, with one study reporting as much as a 45% increase per 2 μg/m^3^ increase of PM2.5 (Wilker et al., 2023).

## Discussion

US Veterans have elevated prevalence of several risk factors for ADRD. Clinicians should implement a cognitive screening pathway for Veterans aged 55 years and older. Initial risk-tiering begins with patient health factors (e.g., hypertension, smoking) and screening assessments including the PHQ-9 (depression), AUDIT-C (alcohol), STOP-BANG (sleep), ACORN (social isolation), and PEG (pain). Veterans identified at high risk for cognitive disorders should undergo cognitive screening (e.g., Mini-Cog or Montreal Cognitive Assessment). Veterans with evidence of cognitive impairment would be referred to memory health or behavioral health programs. Further, based on the positive factors from the initial risk-tiering, patients should be referred for relevant treatment including 12-step programs (substance abuse), sleep medicine, a VA social worker, or pain management. These steps effectively close the loop, ensuring the patient's information and needs are delivered and acted upon. Also, VA providers should make increasing use of StayQuit Coach for smoking cessation, CogSMART for cognitive function and TBI, and the PTSD Coach app for managing symptoms of trauma. Additional programs could build on these models with interventions for physical activity, social engagement, and nutritional assistance. This pathway more effectively detects cognitive decline early, facilitating earlier intervention.

In addition to clinical improvements, there is a lot of opportunity to improve research in this area. First, there is significant variability in methodologies, and high reliance on observational or self-report measures to study risk factor prevalences. Second, it is difficult to establish causal links between risk factors and cognitive decline. These limitations should be addressed by future research. Large scale epidemiological studies with standardized methodologies, definitions, and reporting are needed to accurately compare risk factor prevalence between these populations. Further, established interventions like those for hearing impairment and smoking should be studied in the context of ADRD to evaluate long-term benefits. Additionally, studies are needed to assess the effectiveness of novel and emerging strategies including lifestyle, social, and cognitive interventions. These should include Veteran and non-veteran cohorts, as well as longitudinal elements.

While it is clear that Veterans have unique risk factor profiles, the subject is still emerging. It is possible that these risk factors have secondary or mitigating relationships that influence a Veteran's risk of cognitive decline. However, it is ill-advised to simply sum their effects because several risk factors cluster (e.g., depression, social isolation, sleep disturbance) and share causal pathways. Additionally, there are differences in risk factor prevalence within subgroups of the Veteran population. Female, racial and ethnic minority groups, rural communities, and Veterans from different service eras have different risk factor profiles. For example, estrogen may play a role in females developing ADRD more often than males (Kang and Grodstein, 2012; Yaffe et al., 2000) and Black and Hispanic minorities have greater prevalence of cardiovascular disease and diabetes (2024 Alzheimer's disease facts and figures, 2024; Daniel et al., 2023). Finally, care access patterns of subgroups must also be considered. For example, those living in rural communities may have transportation limitations (Syed et al., 2013; Wiese et al., 2023) and Veterans of different service eras often experience delays in eligibility related to unique exposures (e.g., Agent Orange; Chari et al., 2024). Combinations of risk factors, groups, and subgroups currently make accurate estimates of prevalence difficult, but these estimates must be pursued as this field advances.

## Conclusion

This perspective highlights the importance of addressing the unique risk factor profile of Veterans for the development of ADRD. New efforts to reduce the effect of risk factors for ADRD could improve outcomes and reduce burdens to Veterans and their caregivers. While the impact of addressing these risk factors is difficult to calculate precisely, an estimated 45% of dementia cases are attributed to modifiable risk factors (Livingston et al., 2024). This suggests a significant opportunity for improving health outcomes. The Alzheimer's Association estimates $7 trillion could be saved by the year 2050 by detecting Alzheimer's Disease in the stage of MCI (Porsteinsson et al., 2021). Conducting standardized cognitive screening at an earlier age and on a regular basis may be a key to achieving these goals, because it would allow for earlier detection and intervention, as well as better understanding of risk profiles.

## Data Availability

The original contributions presented in the study are included in the article/supplementary material, further inquiries can be directed to the corresponding author.

## References

[B1] 2024 Alzheimer's disease facts and figures (2024). Alzheimer's Association Report 20, 3708–3821. doi: 10.1002/alz.13809 PMC11095490 38689398

[B2] Agaku I. T. King B. A. Dube S. R. Centers for Disease Control and Prevention (CDC) (2014). Current cigarette smoking among adults – United States, 2005–2012. Morb. Mortal. Wkly. Rep. 63, 29–34. Available online at: https://www.cdc.gov/mmwr/preview/mmwrhtml/mm6302a2.htm (Accessed April 9, 2025). 24430098 PMC4584648

[B3] Alexander M. Ray M. A. Hébert J. R. Youngstedt S. D. Zhang H. Steck S. E. . (2016). The national veteran sleep disorder study: descriptive epidemiology and secular trends, 2000–2010. Sleep 39, 1399–1410. doi: 10.5665/sleep.5972 27091538 PMC4909622

[B4] Barnes D. E. Byers A. L. Gardner R. C. Seal K. H. Boscardin W. J. Yaffe K. . (2018). Association of mild traumatic brain injury with and without loss of consciousness with dementia in US military veterans. JAMA Neurol. 75:1055. doi: 10.1001/jamaneurol.2018.0815 29801145 PMC6143113

[B5] Barnes D. E. Yaffe K. (2011). The projected effect of risk factor reduction on Alzheimer's disease prevalence. Lancet Neurol. 10, 819–828. doi: 10.1016/S1474-4422(11)70072-2 21775213 PMC3647614

[B6] Benoit S. R. Hora I. Albright A. L. Gregg E. W. (2019). New directions in incidence and prevalence of diagnosed diabetes in the USA. BMJ Open Diab. Res. Care 7:e000657. doi: 10.1136/bmjdrc-2019-000657 31245008 PMC6557467

[B7] Bhattarai J. “Jackie,” Oehlert M. E. Multon K. D. Sumerall S. W. (2019). Dementia and cognitive impairment among U.S. veterans with a history of MDD or PTSD: a retrospective cohort study based on sex and race. J. Aging Health 31, 1398–1422. doi: 10.1177/0898264318781131 29900802

[B8] Boersma P. Cohen R. Zelaya C. Moy E. (2021). Multiple chronic conditions among veterans and nonveterans: United States, 2015–2018 *Natl. Health Stat. Report* 1–13. doi: 10.15620/cdc:101659 33663648

[B9] Breland J. Y. Phibbs C. S. Hoggatt K. J. Washington D. L. Lee J. Haskell S. . (2017). The obesity epidemic in the veterans health administration: prevalence among key populations of women and men veterans. J. Gen. Intern. Med. 32, 11–17. doi: 10.1007/s11606-016-3962-1 28271422 PMC5359156

[B10] Brown D. W. (2010). Smoking prevalence among US veterans. J. Gen. Intern. Med. 25, 147–149. doi: 10.1007/s11606-009-1160-0 19894079 PMC2837499

[B11] Carretero O. A. Oparil S. (2000). Essential hypertension. Part 1: definition and etiology. Circulation 101, 329–335. doi: 10.1161/01.CIR.101.3.329 10645931

[B12] Carta M. G. Cossu G. Pintus E. Zaccheddu R. Callia O. Conti G. . (2021). Moderate exercise improves cognitive function in healthy elderly people: results of a randomized controlled trial. Clin. Pract. Epidemiol. Ment. Health 17, 75–80. doi: 10.2174/1745017902117010075 34733346 PMC8493830

[B13] Centers for Disease Control and Prevention (2023). Estimated Hypertension Prevalence, Treatment, and Control Among U.S. Adults. Million Hearts. Atlanta, GA: U.S. Department of Health and Human Services. Available online at: https://millionhearts.hhs.gov/data-reports/hypertension-prevalence.html (Accessed November 7, 2025).

[B14] Chakrabarti R. Garcia D. Pinkovskiy M. (2023). Do Veterans Face Disparities in Higher Education, Health, and Housing? Federal Reserve Bank of New York. Liberty Street Economics. Available online at: https://libertystreeteconomics.newyorkfed.org/2023/05/do-veterans-face-disparities-in-the-labor-market-and-what-accounts-for-them/ (Accessed May 23, 2025).

[B15] Chari R. Salazar H. M. Skrabala L. (2024). Lessons from 9/11 for supporting veterans exposed to military environmental hazards: veterans' issues in focus. Rand Health Q 11:4. Available online at: https://www.rand.org/pubs/periodicals/health-quarterly/issues/v11/n4/04.html (Accessed April 17, 2025). 39346103 10.7249/rhq-11-04-04 PMC11425928

[B16] Chooi Y. C. Ding C. Magkos F. (2019). The epidemiology of obesity. Metabolism 92, 6–10. doi: 10.1016/j.metabol.2018.09.005 30253139

[B17] Civeira F. Arca M. Cenarro A. Hegele R. A. (2022). A mechanism-based operational definition and classification of hypercholesterolemia. J. Clin. Lipidol. 16, 813–821. doi: 10.1016/j.jacl.2022.09.006 36229375

[B18] Clouston S. A. P. Smith D. M. Mukherjee S. Zhang Y. Hou W. Link B. G. . (2020). Education and cognitive decline: an integrative analysis of global longitudinal studies of cognitive aging. J. Gerontol. Ser. B 75, e151–e160. doi: 10.1093/geronb/gbz053 31059564 PMC7424268

[B19] Crimmins E. M. Saito Y. Kim J. K. Zhang Y. S. Sasson I. Hayward M. D. . (2018). Educational differences in the prevalence of dementia and life expectancy with dementia: changes from 2000 to 2010. J. Gerontol. Ser. B 73, S20–S28. doi: 10.1093/geronb/gbx135 29669097 PMC6019027

[B20] Daly M. Sutin A. R. Robinson E. (2021). Depression reported by US adults in 2017–2018 and March and April 2020. J. Affect. Disord. 278, 131–135. doi: 10.1016/j.jad.2020.09.065 32956962 PMC7490280

[B21] Daniel E. V. Kleiman M. J. Galvin J. E. (2023). Exploring reasons for differential vulnerability and Alzheimer's disease risk in racial and ethnic minorities. J. Alzheimers Dis. 91, 495–506. doi: 10.3233/JAD-220959 36442203 PMC10515192

[B22] DeBeer B. B. Davidson D. Meyer E. C. Kimbrel N. A. Gulliver S. B. Morissette S. B. . (2017). The association between toxic exposures and chronic multisymptom illness in veterans of the wars of Iraq and Afghanistan. J. Occup. Environ. Med. 59, 54–60. doi: 10.1097/JOM.0000000000000922 28045798 PMC5556390

[B23] Department of Defense (2023). 2022 Demographics profile of the military community. Washington, DC: Office of the Deputy Assistant Secretary of Defense for Military Community and Family Policy. Available online at: https://download.militaryonesource.mil/12038/MOS/Reports/2022-demographics-report.pdf (Accessed May 22, 2025).

[B24] Donovan N. J. Blazer D. (2020). Social isolation and loneliness in older adults: review and commentary of a national academies report. Am. J. Geriatr. Psychiatry 28, 1233–1244. doi: 10.1016/j.jagp.2020.08.005 32919873 PMC7437541

[B25] Eibner C. Krull H. Brown K. M. Cefalu M. Mulcahy A. W. Pollard M. . (2016). Current and projected characteristics and unique health care needs of the patient population served by the department of veterans affairs. Rand Health Q 5:13. Available online at: https://www.rand.org/pubs/periodicals/health-quarterly/issues/v5/n4/13.html 28083423 10.7249/rhq-05-04-13 PMC5158228

[B26] Elser H. Horváth-Puhó E. Gradus J. L. Smith M. L. Lash T. L. Glymour M. M. . (2023). Association of early-, middle-, and late-life depression with incident dementia in a Danish cohort. JAMA Neurol. 80:949. doi: 10.1001/jamaneurol.2023.2309 37486689 PMC10366950

[B27] Faestel P. M. Littell C. T. Vitiello M. V. Forsberg C. W. Littman A. J. (2013). Perceived insufficient rest or sleep among veterans: behavioral risk factor surveillance system 2009. J. Clin. Sleep Med. 9, 577–584. doi: 10.5664/jcsm.2754 23772191 PMC3659378

[B28] Feigin V. L. Theadom A. Barker-Collo S. Starkey N. J. McPherson K. Kahan M. . (2013). Incidence of traumatic brain injury in New Zealand: a population-based study. Lancet Neurol. 12, 53–64. doi: 10.1016/S1474-4422(12)70262-4 23177532

[B29] Finnegan A. Randles R. (2023). Prevalence of common mental health disorders in military veterans: using primary healthcare data. BMJ Mil. Health 169, 523–528. doi: 10.1136/bmjmilitary-2021-002045 35042763 PMC10715474

[B30] Flegal K. M. Kruszon-Moran D. Carroll M. D. Fryar C. D. Ogden C. L. (2016). Trends in obesity among adults in the United States, 2005 to 2014. JAMA 315, 2284–2291. doi: 10.1001/jama.2016.6458 27272580 PMC11197437

[B31] Fung C. H. Martin J. L. Dzierzewski J. M. Jouldjian S. Josephson K. Park M. . (2013). Prevalence and symptoms of occult sleep disordered breathing among older veterans with insomnia. J. Clin. Sleep Med. 09, 1173–1178. doi: 10.5664/jcsm.3162 24235899 PMC3805803

[B32] Gardner R. C. Bahorik A. Kornblith E. S. Allen I. E. Plassman B. L. Yaffe K. . (2023). Systematic review, meta-analysis, and population attributable risk of dementia associated with traumatic brain injury in civilians and veterans. J. Neurotrauma. 40, 620–634. doi: 10.1089/neu.2022.0041 36305374 PMC10325813

[B33] Goldstein L. A. Bernhard P. A. Hoffmire C. A. Schneiderman A. Maguen S. (2025). Prevalence of obstructive sleep apnea among veterans and nonveterans. Am. J. Health Promot. 39, 215–223. doi: 10.1177/08901171241273443 39136615

[B34] Gould C. E. Rideaux T. Spira A. P. Beaudreau S. A. (2015). Depression and anxiety symptoms in male veterans and non-veterans: the health and retirement study. Int. J. Geriatr. Psychiatry 30, 623–630. doi: 10.1002/gps.4193 25145943 PMC4336840

[B35] Günak M. M. Billings J. Carratu E. Marchant N. L. Favarato G. Orgeta V. . (2020). Post-traumatic stress disorder as a risk factor for dementia: systematic review and meta-analysis. Br. J. Psychiatry 217, 600–608. doi: 10.1192/bjp.2020.150 32933591

[B36] Hales C. M. Carroll M. D. Fryar C. D. Ogden C. L. (2020). Prevalence of Obesity and Severe Obesity Among Adults: United States, 2017–2018. National Center for Health Statistics Data Brief, 1–8. Available online at: https://www.cdc.gov/nchs/products/databriefs/db360.htm (Accessed May 29, 2024). 32487284

[B37] Hankin C. S. Spiro A. Miller D. R. Kazis L. (1999). Mental disorders and mental health treatment among U.S. department of veterans affairs outpatients: the veterans health study. Am. J. Psychiatry 156, 1924–1930. doi: 10.1176/ajp.156.12.1924 10588406

[B38] Heckman J. J. LaFontaine P. A. (2010). The American high school graduation rate: trends and levels. Rev. Econ. Stat. 92, 244–262. doi: 10.1162/rest.2010.12366 20625528 PMC2900934

[B39] Hoerster K. D. Lehavot K. Simpson T. McFall M. Reiber G. Nelson K. M. . (2012). Health and health behavior differences: US Military, veteran, and civilian men. Am. J. Prev. Med. 43, 483–489. doi: 10.1016/j.amepre.2012.07.029 23079170

[B40] Hoisington A. J. Stearns-Yoder K. A. Kovacs E. J. Postolache T. T. Brenner L. A. (2024). Airborne exposure to pollutants and mental health: a review with implications for United States veterans. Curr. Environ. Health Rep. 11, 168–183. doi: 10.1007/s40572-024-00437-8 38457036 PMC12070290

[B41] Jackson S. L. Long Q. Rhee M. K. Olson D. E. Tomolo A. M. Cunningham S. A. . (2015). Weight loss and incidence of diabetes with the veterans health administration MOVE! lifestyle change programme: an observational study. Lancet Diabetes Endocrinol. 3, 173–180. doi: 10.1016/S2213-8587(14)70267-0 25652129 PMC4401476

[B42] Jeon K. H. Han K. Jeong S-. M. Park J. Yoo J. E. Yoo J. . (2023). Changes in alcohol consumption and risk of dementia in a nationwide cohort in South Korea. JAMA Netw. Open 6:e2254771. doi: 10.1001/jamanetworkopen.2022.54771 36745453 PMC12549098

[B43] Kang J. H. Grodstein F. (2012). Postmenopausal hormone therapy, timing of initiation, APOE and cognitive decline. Neurobiol. Aging 33, 1129–1137. doi: 10.1016/j.neurobiolaging.2010.10.007 21122949 PMC3483632

[B44] Kannan V. D. Veazie P. J. (2023). US trends in social isolation, social engagement, and companionship—nationally and by age, sex, race/ethnicity, family income, and work hours, 2003–2020. SSM—*Popul. Health* 21:101331. doi: 10.1016/j.ssmph.2022.101331 PMC9811250 36618547

[B45] Kivimäki M. Singh-Manoux A. Pentti J. Sabia S. Nyberg S. T. Alfredsson L. . (2019). Physical inactivity, cardiometabolic disease, and risk of dementia: an individual-participant meta-analysis. BMJ 365:l1495. doi: 10.1136/bmj.l1495 30995986 PMC6468884

[B46] Kornblith E. S. Yaffe K. Langa K. M. Gardner R. C. (2020). Prevalence of lifetime history of traumatic brain injury among older male veterans compared with civilians: a nationally representative study. J. Neurotrauma. 37, 2680–2685. doi: 10.1089/neu.2020.7062 32762279 PMC7869884

[B47] Kuiper J. S. Zuidersma M. Oude Voshaar R. C. Zuidema S. U. Van Den Heuvel E. R. Stolk R. P. . (2015). Social relationships and risk of dementia: a systematic review and meta-analysis of longitudinal cohort studies. Ageing Res. Rev. 22, 39–57. doi: 10.1016/j.arr.2015.04.006 25956016

[B48] Littman A. J. Forsberg C. W. Boyko E. J. (2013). Associations between compulsory physical activity during military service and activity in later adulthood among male veterans compared with nonveterans. J. Phys. Act. Health 10, 784–791. doi: 10.1123/jpah.10.6.784 23074136

[B49] Liu Y. Sayam S. Shao X. Wang K. Zheng S. Li Y. . (2017). Prevalence of and trends in diabetes among veterans, United States, 2005–2014. Prev. Chronic. Dis. 14:E135. doi: 10.5888/pcd14.170230 29240552 PMC5737977

[B50] Livingston G. Huntley J. Liu K. Y. Costafreda S. G. Selbæk G. Alladi S. . (2024). Dementia prevention, intervention, and care: 2024 report of the lancet standing commission. Lancet 404, 572–628. doi: 10.1016/S0140-6736(24)01296-0 39096926

[B51] Livingston G. Huntley J. Sommerlad A. Ames D. Ballard C. Banerjee S. . (2020). Dementia prevention, intervention, and care: 2020 report of the lancet commission. Lancet 396, 413–446. doi: 10.1016/S0140-6736(20)30367-6 32738937 PMC7392084

[B52] Lucas J. W. Zelaya C. E. (2020). Hearing Difficulty, Vision Trouble, and Balance Problems Among Male Veterans and Nonveterans. National Health Statistics Reports, National Center for Health Statistics, 1–8. 32600517

[B53] Ma Y. Ajnakina O. Steptoe A. Cadar D. (2020). Higher risk of dementia in English older individuals who are overweight or obese. Int. J. Epidemiol. 49, 1353–1365. doi: 10.1093/ije/dyaa099 32575116 PMC7660153

[B54] Madans J. H. Weeks J. D. Elgaddal N. (2021). Hearing Difficulties Among Adults: United States, 2019 (No. 414). National Center for Health Statistics, 1–8. doi: 10.15620/cdc:107540 34319870

[B55] Mannes Z. L. Stohl M. Fink D. S. Olfson M. Keyes K. M. Martins S. S. . (2022). Non-pharmacological treatment for chronic pain in US veterans treated within the veterans health administration: implications for expansion in US healthcare systems. J. Gen. Intern. Med. 37, 3937–3946. doi: 10.1007/s11606-021-07370-8 35048300 PMC8769678

[B56] Martinez S. Yaffe K. Li Y. Byers A. L. Peltz C. B. Barnes D. E. . (2021). Agent orange exposure and dementia diagnosis in US veterans of the Vietnam era. JAMA Neurol. 78, 473–477. doi: 10.1001/jamaneurol.2020.5011 33492338 PMC7835948

[B57] Moradi Y. Dowran B. Sepandi M. (2021). The global prevalence of depression, suicide ideation, and attempts in the military forces: a systematic review and meta-analysis of cross sectional studies. BMC Psychiatry 21:510. doi: 10.1186/s12888-021-03526-2 34654386 PMC8520236

[B58] Mustafa M. Erokwu N. Ebose I. Strohl K. (2005). Sleep problems and the risk for sleep disorders in an outpatient veteran population. Sleep Breath 9, 57–63. doi: 10.1007/s11325-005-0016-z 15875229

[B59] Nahin R. L. Feinberg T. Kapos F. P. Terman G. W. (2023). Estimated rates of incident and persistent chronic pain among US adults, 2019–2020. JAMA Netw. Open 6:e2313563. doi: 10.1001/jamanetworkopen.2023.13563 37191961 PMC10189566

[B60] Nandi A. Counts N. Chen S. Seligman B. Tortorice D. Vigo D. . (2022). Global and regional projections of the economic burden of Alzheimer's disease and related dementias from 2019 to 2050: a value of statistical life approach. eClinicalMedicine 51:101580. doi: 10.1016/j.eclinm.2022.101580 35898316 PMC9310134

[B61] National Center for Education Statistics (2024). High school graduation rates. Condition of Education. Washington, DC: U.S. Department of Education, Institute of Education Sciences. Available online at: https://nces.ed.gov/programs/coe/indicator/coi/high-school-graduation-rates (Accessed November 7, 2025).

[B62] National Institute on Deafness and Other Communication Disorders (2024). Quick statistics about hearing, balance, & dizziness. Bethesda, MD: National Institutes of Health. Available online at: https://www.nidcd.nih.gov/health/statistics/quick-statistics-hearing (Accessed April 3, 2025).

[B63] Nguyen X. T. Ho Y. Li Y. Song R. J. Leung K. H. Rahman S. U. . (2023). Serum cholesterol and impact of age on coronary heart disease death in more than 4 million veterans. J. Am. Heart Assoc. 12:e030496. doi: 10.1161/JAHA.123.030496 37889207 PMC10727410

[B64] Nieh C. Mancuso J. D. Powell T. M. Welsh M. M. Gackstetter G. D. Hooper T. I. . (2021). Cigarette smoking patterns among U.S. military service members before and after separation from the military. PLOS ONE 16:e0257539. doi: 10.1371/journal.pone.0257539 34606513 PMC8489722

[B65] Norton S. Matthews F. E. Barnes D. E. Yaffe K. Brayne C. (2014). Potential for primary prevention of Alzheimer's disease: an analysis of population-based data. Lancet Neurol. 13, 788–794. doi: 10.1016/S1474-4422(14)70136-X 25030513

[B66] Odani S. Agaku I. T. Graffunder C. M. Tynan M. A. Armour B. S. (2018). Tobacco product use among military veterans—United States, 2010–2015. MMWR Morb. Mortal. Wkly. Rep. 67, 7–12. doi: 10.15585/mmwr.mm6701a2 29324732 PMC5769800

[B67] Ogden C. L. Fryar C. D. Martin C. B. Freedman D. S. Carroll M. D. Gu Q. . (2020). Trends in obesity prevalence by race and hispanic origin–1999-2000 to 2017-2018. JAMA 324, 1208–1210. doi: 10.1001/jama.2020.14590 32857101 PMC7455882

[B68] Penninkilampi R. Casey A-. N. Singh M. F. Brodaty H. (2018). The association between social engagement, loneliness, and risk of dementia: a systematic review and meta-analysis. J. Alzheimers Dis. 66, 1619–1633. doi: 10.3233/JAD-180439 30452410

[B69] Phillips E. Wang T. W. Husten C. G. Corey C. G. Apelberg B. J. Jamal A. . (2017). Tobacco product use among adults—United States, 2015. MMWR Morb. Mortal Wkly. Rep. 66, 1209–1215. doi: 10.15585/mmwr.mm6644a2 29121001 PMC5679591

[B70] Plassman B. L. Havlik R. J. Steffens D. C. Helms M. J. Newman T. N. Drosdick D. . (2000). Documented head injury in early adulthood and risk of Alzheimer's disease and other dementias. Neurology 55, 1158–1166. doi: 10.1212/WNL.55.8.1158 11071494

[B71] Porsteinsson A. P. Isaacson R. S. Knox S. Sabbagh M. N. Rubino I. (2021). Diagnosis of early Alzheimer's disease: clinical practice in 2021. J. Prev. Alzheimers Dis. 8, 371–386. doi: 10.14283/jpad.2021.23 34101796 PMC12280795

[B72] Powell W. R. Vilen L. Zuelsdorff M. Goutman S. A. Salamat S. Rissman R. A. . (2023). Association between military service and Alzheimer's disease neuropathology at autopsy. Alzheimers Dement. 20, 1468–1474. doi: 10.1002/alz.13520 37965965 PMC10917028

[B73] Qi X. Zhu Z. Luo H. Schwartz M. D. Wu B. (2024). Age at diagnosis of diabetes, obesity, and the risk of dementia among adult patients with type 2 diabetes. PLOS ONE 19:e0310964. doi: 10.1371/journal.pone.0310964 39535979 PMC11559992

[B74] Qureshi S. U. Kimbrell T. Pyne J. M. Magruder K. M. Hudson T. J. Petersen N. J. . (2010). Greater prevalence and incidence of dementia in older veterans with posttraumatic stress disorder: [See editorial comments by Dr. Soo Borson, pp 1797-1798]. J. Am. Geriatr. Soc. 58, 1627–1633. doi: 10.1111/j.1532-5415.2010.02977.x 20863321

[B75] Rikard S. M. Strahan A. E. Schmit K. M. Jr G. P. G. (2023). Chronic pain among adults—United States, 2019–2021. MMWR Morb. Mortal Wkly. Rep. 72, 379–385. doi: 10.15585/mmwr.mm7215a1 37053114 PMC10121254

[B76] Rolen E. (2017). A Closer Look at Veterans in the Labor Force, Career Outlook. Washington, DC: U.S. Bureau of Labor Statistics. Available online at: https://www.bls.gov/careeroutlook/2017/article/veterans.htm (Accessed November 12, 2025).

[B77] Rondón Bernard J. E. (2018). Depression: a review of its definition. MOJ Addict. Med. Ther. 5, 6–7. doi: 10.15406/mojamt.2018.05.00082

[B78] Sabia S. Fayosse A. Dumurgier J. Dugravot A. Akbaraly T. Britton A. . (2018). Alcohol consumption and risk of dementia: 23 year follow-up of Whitehall II cohort study. BMJ 362:k2927. doi: 10.1136/bmj.k2927 30068508 PMC6066998

[B79] Saczynski J. S. Pfeifer L. A. Masaki K. Korf E. S. C. Laurin D. White L. . (2006). The effect of social engagement on incident dementia. Am. J. Epidemiol. 163, 433–440. doi: 10.1093/aje/kwj061 16410348

[B80] Sáez De Asteasu M. L. Martínez-Velilla N. Zambom-Ferraresi F. Casas-Herrero Á. Izquierdo M. (2017). Role of physical exercise on cognitive function in healthy older adults: A systematic review of randomized clinical trials. Ageing Res. Rev. 37, 117–134. doi: 10.1016/j.arr.2017.05.007 28587957

[B81] Schnurr P. P. Lunney C. A. Bovin M. J. Marx B. P. (2009). Posttraumatic stress disorder and quality of life: extension of findings to veterans of the wars in Iraq and Afghanistan. Clin. Psychol. Rev. 29, 727–735. doi: 10.1016/j.cpr.2009.08.006 19744758

[B82] Sharp E. S. Gatz M. (2011). Relationship between education and dementia: an updated systematic review. Alzheimer Dis. Assoc. Disord. 25, 289–304. doi: 10.1097/WAD.0b013e318211c83c 21750453 PMC3193875

[B83] Shi L. Chen S-. J. Ma M-. Y. Bao Y-. P. Han Y. Wang Y-. M. . (2018). Sleep disturbances increase the risk of dementia: a systematic review and meta-analysis. Sleep Med. Rev. 40, 4–16. doi: 10.1016/j.smrv.2017.06.010 28890168

[B84] Skalny A. V. Aschner M. Bobrovnitsky I. P. Chen P. Tsatsakis A. Paoliello M. M. B. . (2021). Environmental and health hazards of military metal pollution. Environ. Res. 201:111568. doi: 10.1016/j.envres.2021.111568 34174260

[B85] Skaria A. P. (2022). The economic and societal burden of Alzheimer disease: managed care considerations. Am. J. Manag. Care 28, S188–S196. doi: 10.37765/ajmc.2022.89236 36197132

[B86] Smushkin G. Vella A. (2010). What is type 2 diabetes? Medicine (Baltimore) 38, 597–601. doi: 10.1016/j.mpmed.2010.08.008 PMC3073595 21151710

[B87] Soh Y. Whitmer R. A. Mayeda E. R. Glymour M. M. Peterson R. L. Eng C. W. . (2023). State-level indicators of childhood educational quality and incident dementia in older Black and White adults. JAMA Neurol. 80:352. doi: 10.1001/jamaneurol.2022.5337 36780143 PMC9926357

[B88] Substance Abuse and Mental Health Services Administration (2022). Key substance use and mental health indicators in the United States: Results from the 2021 National Survey on Drug Use and Health (HHS Publication No. PEP22-07-01-005, NSDUH Series H-57). Rockville, MD: Center for Behavioral Health Statistics and Quality, Substance Abuse and Mental Health Services Administration. Available online at: https://www.samhsa.gov/data/report/2021-nsduh-annual-national-report (Accessed November 7, 2025).

[B89] Syed S. T. Gerber B. S. Sharp L. K. (2013). Traveling towards disease: transportation barriers to health care access. J. Community Health 38, 976–993. doi: 10.1007/s10900-013-9681-1 23543372 PMC4265215

[B90] Taljaard D. S. Olaithe M. Brennan-Jones C. G. Eikelboom R. H. Bucks R. S. (2016). The relationship between hearing impairment and cognitive function: a meta-analysis in adults. Clin. Otolaryngol. 41, 718–729. doi: 10.1111/coa.12607 26670203

[B91] Taylor K. A. Kapos F. P. Sharpe J. A. Kosinski A. S. Rhon D. I. Goode A. P. . (2024). Seventeen-year national pain prevalence trends among U.S. military veterans. J. Pain 25:104420. doi: 10.1016/j.jpain.2023.11.003 37952861 PMC11184511

[B92] Teeters J. Lancaster C. Brown D. Back S. (2017). Substance use disorders in military veterans: prevalence and treatment challenges. Subst. Abuse Rehabil. 8, 69–77. doi: 10.2147/SAR.S116720 28919834 PMC5587184

[B93] Thivel D. Tremblay A. Genin P. M. Panahi S. Rivière D. Duclos M. . (2018). Physical activity, inactivity, and sedentary behaviors: definitions and implications in occupational health. Front. Public Health 6:288. doi: 10.3389/fpubh.2018.00288 30345266 PMC6182813

[B94] Tian J. Jones G. Lin X. Zhou Y. King A. Vickers J. . (2023). Association between chronic pain and risk of incident dementia: findings from a prospective cohort. BMC Med. 21:169. doi: 10.1186/s12916-023-02875-x 37143042 PMC10161483

[B95] Treede R-. D. Rief W. Barke A. Aziz Q. Bennett M. I. Benoliel R. . (2019). Chronic pain as a symptom or a disease: the IASP classification of chronic pain for the international classification of diseases (ICD-11). Pain 160, 19–27. doi: 10.1097/j.pain.0000000000001384 30586067

[B96] Trembley J. H. Barach P. Tomáška J. M. Poole J. T. Ginex P. K. Miller R. F. . (2024). Current understanding of the impact of United States military airborne hazards and burn pit exposures on respiratory health. Part. Fibre Toxicol. 21:43. doi: 10.1186/s12989-024-00606-5 39434148 PMC11492460

[B97] Tsao C. W. Aday A. W. Almarzooq Z. I. Anderson C. A. M. Arora P. Avery C. L. . (2023). Heart disease and stroke statistics?2023 update: A report from the American Heart Association. Circulation. 147, e93–e621. doi: 10.1161/CIR.0000000000001123 36695182 PMC12135016

[B98] Veitch D. Friedl K. Weiner M. (2013). Military risk factors for cognitive decline, dementia and Alzheimer's disease. Curr. Alzheimer Res. 10, 907–930. doi: 10.2174/15672050113109990142 23906002

[B99] Vespa J. (2023). Aging Veterans: America's Veteran Population in Later Life (No. ACS-54), American Community Survey Reports. Washington, DC: U.S. Census Bureau. Available online at: https://www.census.gov/library/publications/2023/acs/acs-54.html (Accessed March 25, 2025).

[B100] Wagner T. H. Harris K. M. Federman B. Dai L. Luna Y. Humphreys K. . (2007). Prevalence of substance use disorders among veterans and comparable nonveterans from the national survey on drug use and health. Psychol. Serv. 4:149. doi: 10.1037/1541-1559.4.3.149

[B101] Walker A. Chapin B. Abisambra J. DeKosky S. T. (2022). Association between single moderate to severe traumatic brain injury and long-term tauopathy in humans and preclinical animal models: a systematic narrative review of the literature. Acta Neuropathol. Commun. 10:13. doi: 10.1186/s40478-022-01311-0 35101132 PMC8805270

[B102] Wang L. Li X. Wang Z. Bancks M. P. Carnethon M. R. Greenland P. . (2021). Trends in prevalence of diabetes and control of risk factors in diabetes among US adults, 1999-2018. JAMA 326, 704–716. doi: 10.1001/jama.2021.9883 34170288 PMC8233946

[B103] Washington D. L. (ed.). (2022). National Veteran Health Equity Report 2021. Focus on Veterans Health Administration Patient Experience and Health Care Quality. Washington, DC: VHA Office of Health Equity. Available online at: https://www.va.gov/HEALTHEQUITY/docs/NVHER_2021_Report_508_Conformant.pdf (Accessed November 5, 2025).

[B104] Wee J. Sukudom S. Bhat S. Marklund M. Peiris N. J. Hoyos C. M. . (2023). The relationship between midlife dyslipidemia and lifetime incidence of dementia: a systematic review and meta-analysis of cohort studies. Alzheimers Dement. Diagn. Assess. Dis. Monit. 15:e12395. doi: 10.1002/dad2.12395 36911359 PMC9993469

[B105] Wiese L. A. K. Gibson A. Guest M. A. Nelson A. R. Weaver R. Gupta A. . (2023). Global rural health disparities in Alzheimer's disease and related dementias: state of the science. Alzheimers Dement. 19, 4204–4225. doi: 10.1002/alz.13104 37218539 PMC10524180

[B106] Wilker E. H. Osman M. Weisskopf M. G. (2023). Ambient air pollution and clinical dementia: systematic review and meta-analysis. BMJ. 381:e071620. doi: 10.1136/bmj-2022-071620 37019461 PMC10498344

[B107] Wisco B. E. Nomamiukor F. O. Marx B. P. Krystal J. H. Southwick S. M. Pietrzak R. H. . (2022). Posttraumatic stress disorder in US military veterans: results from the 2019–2020 national health and resilience in veterans study. J. Clin. Psychiatry. 83:20m14029. doi: 10.4088/JCP.20m14029 35192748

[B108] Wong R. Lovier M. A. (2023). Sleep disturbances and dementia risk in older adults: findings from 10 years of national U.S. prospective data. Am. J. Prev. Med. 64, 781–787. doi: 10.1016/j.amepre.2023.01.008 36707315

[B109] Xue M. Xu W. Ou Y-. N. Cao X-. P. Tan M-. S. Tan L. . (2019). Diabetes mellitus and risks of cognitive impairment and dementia: A systematic review and meta-analysis of 144 prospective studies. Ageing Res. Rev. 55:100944. doi: 10.1016/j.arr.2019.100944 31430566

[B110] Yaffe K. Haan M. Byers A. Tangen C. Kuller L. (2000). Estrogen use, APOE, and cognitive decline: evidence of gene–environment interaction. Neurology 54, 1949–1954. doi: 10.1212/WNL.54.10.1949 10822435

[B111] Yaffe K. Vittinghoff E. Lindquist K. Barnes D. Covinsky K. E. Neylan T. . (2010). Posttraumatic stress disorder and risk of dementia among US veterans. Arch. Gen. Psychiatry 67:608. doi: 10.1001/archgenpsychiatry.2010.61 20530010 PMC2933793

[B112] Yamada M. Wachsmuth J. Sambharia M. Griffin B. R. Swee M. L. Reisinger H. S. . (2023). The prevalence and treatment of hypertension in veterans health administration, assessing the impact of the updated clinical guidelines. J. Hypertens. 41, 995–1002. doi: 10.1097/HJH.0000000000003424 37071434 PMC10158602

[B113] Yan S. Fu W. Wang C. Mao J. Liu B. Zou L. . (2020). Association between sedentary behavior and the risk of dementia: a systematic review and meta-analysis. Transl. Psychiatry 10:112. doi: 10.1038/s41398-020-0799-5 32317627 PMC7174309

[B114] Zelaya C. Dahlhamer J. M. Sun Y. (2020). QuickStats: Percentage of adults aged ≥20 years who had chronic pain, by veteran status and age group?National Health Interview Survey, United States, 2019. MMWR Morb. Mortal. Wkly. Rep. 69:1797. doi: 10.15585/mmwr.mm6947a6 33237896 PMC7727604

[B115] Zhong G. Wang Y. Zhang Y. Guo J. J. Zhao Y. (2015). Smoking is associated with an increased risk of dementia: a meta-analysis of prospective cohort studies with investigation of potential effect modifiers. PLOS ONE 10:e0118333. doi: 10.1371/journal.pone.0118333 25763939 PMC4357455

